# No evidence of locus heterogeneity in familial microcephaly with or without chorioretinopathy, lymphedema, or mental retardation syndrome

**DOI:** 10.1186/s13023-015-0271-4

**Published:** 2015-05-02

**Authors:** Matthieu J Schlögel, Antonella Mendola, Elodie Fastré, Pradeep Vasudevan, Koen Devriendt, Thomy JL de Ravel, Hilde Van Esch, Ingele Casteels, Ignacio Arroyo Carrera, Francesca Cristofoli, Karen Fieggen, Katheryn Jones, Mark Lipson, Irina Balikova, Ami Singer, Maria Soller, María Mercedes Villanueva, Nicole Revencu, Laurence M Boon, Pascal Brouillard, Miikka Vikkula

**Affiliations:** Laboratory of Human Molecular Genetics, de Duve Institute, Université catholique de Louvain, Avenue Hippocrate 74, bte B1.74.06, B-1200 Brussels, Belgium; Department of Clinical Genetics, University Hospitals of Leicester, Leicester Royal Infirmary, Leicester, LE1 5WW UK; Center for Human Genetics, University Hospitals Leuven, KU Leuven, 3000 Leuven, Belgium; Department of Ophthalmology, St Rafael University Hospitals, 3000 Leuven, Belgium; Servicio de Pediatría, Hospital San Pedro de Alcántara, Cáceres, Spain; Division of Human Genetics, University of Cape Town, 7700 Cape Town, South Africa; Medical Genetics, Kaiser Permanente, Sacramento, CA 95815 USA; Department of Ophthalmology, Queen Fabiola Children’s University Hospital (HUDERF), 1020 Brussels, Belgium; Pediatrics and Medical Genetics, Barzilai Medical Center, 78306 Ashkelon, Israel; Department of Clinical Genetics, Lund University Hospital, 221 85 Lund, Sweden; General Hospital of Florencio Varela, Children’s Hospital Dr. Pedro Elizalde and Foundation for Neurological Diseases of Childhood (FLENI), C1270AAN Buenos Aires, Capital Federal Argentina; Center for Human Genetics, Cliniques universitaires Saint-Luc, Université catholique de Louvain, 1200 Brussels, Belgium; Center for Vascular Anomalies, Cliniques universitaires Saint-Luc, Université catholique de Louvain, 1200 Brussels, Belgium; Walloon Excellence in Lifesciences and Biotechnology (WELBIO), Université catholique de Louvain, 1200 Brussels, Belgium

**Keywords:** CDMMR, EG5, FEVR, Gene, Intellectual disability, KIF11, MCLMR, MLCRD, Mutation

## Abstract

**Background:**

Microcephaly with or without chorioretinopathy, lymphedema, or mental retardation syndrome (MCLMR) is a rare autosomal dominant disorder with variable expressivity. It is characterized by mild-to-severe microcephaly, often associated with intellectual disability, ocular defects and lymphedema. It can be sporadic or inherited. Eighty-seven patients have been described to carry a mutation in *KIF11*, which encodes a homotetrameric motor kinesin, EG5.

**Methods:**

We tested 23 unreported MCLMR index patients for *KIF11*. We also reviewed the clinical phenotypes of all our patients as well as of those described in previously published studies.

**Results:**

We identified 14 mutations, 12 of which are novel. We detected mutations in 12 affected individuals, from 6 out of 6 familial cases, and in 8 out of 17 sporadic patients. Phenotypic evaluation of patients (our 26 + 61 earlier published = 87) revealed microcephaly in 91%, eye anomalies in 72%, intellectual disability in 67% and lymphedema in 47% of the patients. Unaffected carriers were rare (4 out of 87: 5%). Family history is not a requisite for diagnosis; 31% (16 out of 52) were *de novo* cases.

**Conclusions:**

All inherited cases, and 50% of sporadic cases of MCLMR are due to germline *KIF11* mutations. It is possible that mosaic *KIF11* mutations cause the remainder of sporadic cases, which the methods employed here were not designed to detect. On the other hand, some of them might have another mimicking disorder and genetic defect, as microcephaly is highly heterogeneous. In aggregate, *KIF11* mutations likely cause the majority, if not all, of MCLMR.

## Background

**Figure 1:**
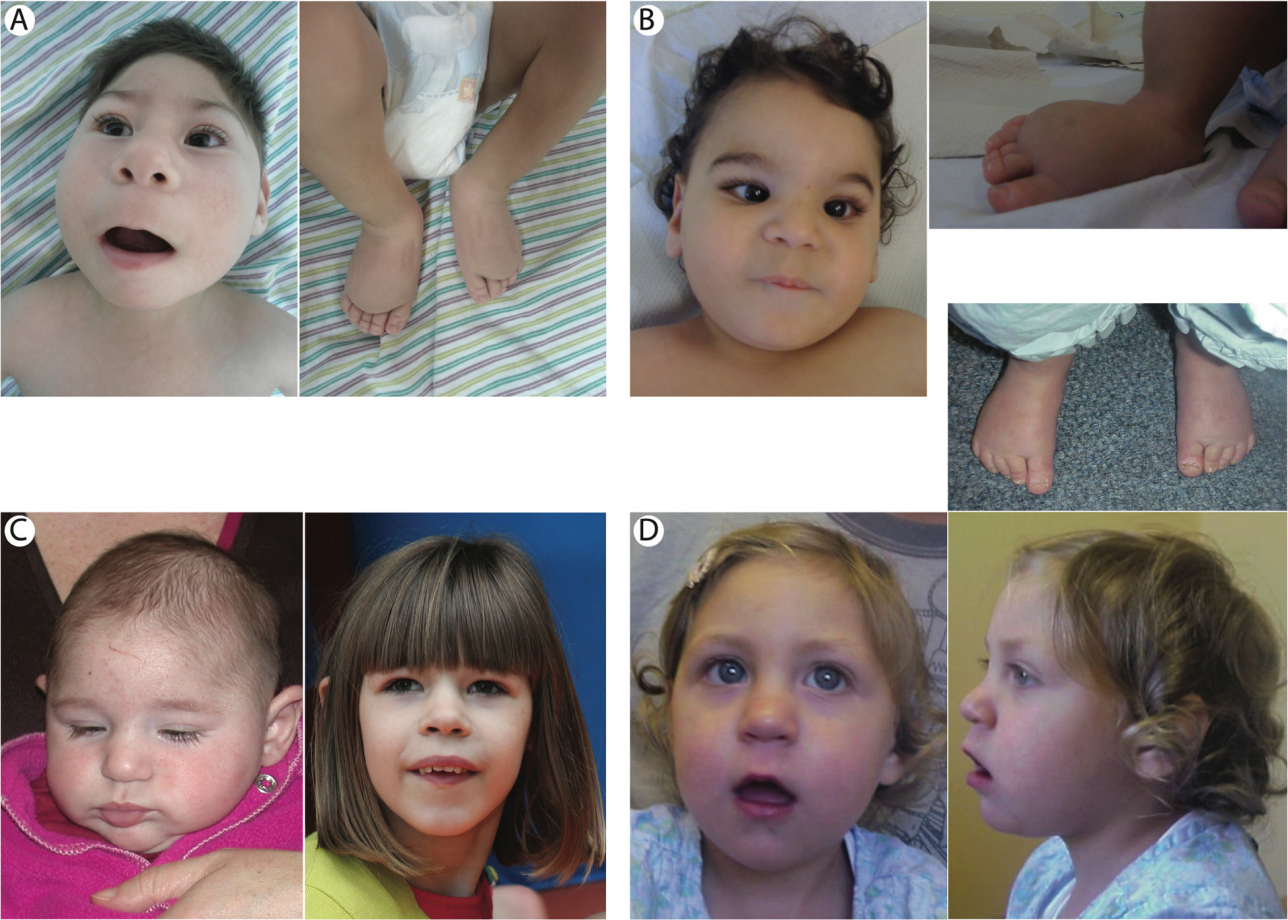
Clinical characteristics of patients. Clinical features of MCMLR (patients I-10 **(A)**, IX-10 **(B)**, X-10 **(C)** and XIV-10 **(D)**. Note, broad nose **(A, B)**, long philtrum **(A, B)**, thin upper lip **(A, D)**, prominent ears **(C, D)** and bilateral pedal lymphedema **(A, B D)**.

Microcephaly with or without chorioretinopathy, lymphedema, or mental retardation syndrome (MCLMR, OMIM 152950, ORPHA2526) can occur as a sporadic or inherited (autosomal dominant) disease [1]. MCLMR is characterized by a broad nose, upslanting palpebral fissures, a rounded nasal tip, a long philtrum, a pointed chin, a thin upper lip, prominent ears, atrial septal defect and lymphedema of the dorsum of the feet (pedal œdema) (Figure 1) [2,3]. Ocular anomalies, the most common of which is chorioretinal dysplasia, require long-term ophthalmic follow-up due to slow development. Cerebral magnetic resonance imaging may reveal a smaller, but usually structurally normal brain [4,5].

Historically, MCLMR syndrome was divided into two distinct entities: microcephaly, lymphedema and chorioretinal dysplasia syndrome (MLCRD), and chorioretinal dysplasia, microcephaly and mental retardation syndrome (CDMMR). The discovery of *KIF11* mutations in patients with MLCRD or CDMMR demonstrated that the phenotypes are part of a single entity with variable penetrance and expressivity [6]. *KIF11* mutations were also discovered in 5 families with familial exudative vitreoretinopathy syndrome (FEVR), which thus belongs to the phenotypic spectrum caused by *KIF11* mutations [7]. Thirty-one different *KIF11* mutations have been described [6-10]. They include missense, nonsense, frameshift and splice-site mutations distributed across the gene, and are thought to cause loss-of-function. No genotype-phenotype correlation has been described. All of the mutations are autosomal-dominant or *de novo*; no autosomal recessive case with a *KIF11* mutation has been described.

**Figure 2:**
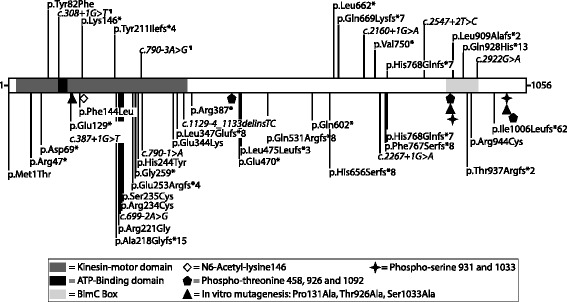
Schematic representation of EG5 (1056 amino acids) with position of mutations causing MCLMR syndrome. Functional domains (colored), *in vitro* mutagenized residues (signs) and post-translationally modified amino acids (signs). Mutations found in this study, top; those published earlier, below (Ostergaard *et al*. 2012, Hazan *et al*. 2012, Jones *et al*. 2013, Mirzaa *et al*. 2014 and Robitaille *et al*. 2014). Splice-site alterations (¶: family II and family V) shown to result in r.211_308del; p.Thr71Argfs*8 and r.789_790insAG; p.Val264Argfs*26, respectively (Figure 4). N.B. positions based on amino acids in Mirzaa *et al*.

*KIF11* is located on *10q23.33* (http://www.ensembl.org) and encodes the kinesin family member 11 (also known as EG5, KNSL1 or TRIP5). EG5 is a 1056-amino-acid protein, which forms homotetrameric kinesin motor complexes. The N-terminal part composes the motor domain (Gly16 – Phe363), containing a microtubule-binding domain that allows sliding along microtubules, an ATP-binding site with ATPase activity (between residues Gly105 and Thr112), and three internal domains that form coiled-coil structures responsible for multimerization into homotetramers (Figure 2). The C-terminal BimC domain also plays a role in multimerization [11].

The aim of this study was to screen *KIF11* in a large cohort of patients with signs of MCLMR in order to identify novel mutations, assess for locus heterogeneity and genotype-phenotype correlations, and better define the clinical phenotype associated with *KIF11* mutations. Both sporadic and familial index patients were included, as well as additional family members when available*.*

## Methods

### Patient recruitment

DNA extracted from blood-samples of patients was collected from different countries (Argentina, Australia, Belgium, Israel, United-Kingdom, Spain and Sweden). Informed consent was obtained for all participants, as approved by the ethical committee of the Medical Faculty at the Université catholique de Louvain, Brussels, Belgium and the respective local committees. DNA was extracted using Wizard genomic DNA purification kit (Promega). Patients V-100, XI-100 and XIII-10 have previously been described in case reports [12-14]. Data on certain phenotypic characteristics, including level of intellectual disability, were not standardized; evaluation by the referring clinician was used. The average age of our patients at the time of conducting the study was 21 years.

### Sanger sequencing of *KIF11*

To screen for *KIF11* mutations, the 22 exons and their corresponding splice-sites were amplified, as described [6]. Purity and size of amplicons were evaluated by agarose gel electrophoresis using GeneRuler™ 100 bp (Thermo Scientific) DNA size ladder. Direct sequencing was performed on a 3130xl Genetic Analyzer (Life Technologies). All sequences were analyzed with CLC Main Workbench 6^©^ (CLC Bio), using reference sequence NG_032580.

### Mutation analysis

Mutations were named according to the guidelines of the Human Genome Variation Society (http://www.hgvs.org/mutnomen/), verified using Mutalyzer (https://mutalyzer.nl/) [15]. The mutations have been submitted to the Vascular Anomaly and Lymphedema Mutation Database (www.icp.ucl.ac.be/vikkula/VAdb). The dbSNP137 (http://www.ncbi.nlm.nih.gov/projects/SNP/) [16], 1000Genomes (http://browser.1000genomes.org), ESP6500 (http://evs.gs.washington.edu/EVS/), and GoNL databases (http://www.nlgenome.nl) of genomic variants were searched for each mutation identified, in order to eliminate any polymorphisms. The effects of amino acid changes on protein function were predicted using PolyPhen-2 (http://genetics.bwh.harvard.edu/pph2/) [17] and SIFT (http://sift.jcvi.org/www/SIFT_enst_submit.html) [18]. MutationTaster (http://www.mutationtaster.org/) [19] was used to detect potential splice-site changes. When DNA from additional family members was available, co-segregation of mutation with disease was assessed. If the mutation was not identified in either parent, the Powerplex HS16 genotyping kit (Promega) on a 3130xl Genetic Analyzer (Life Technologies) was used to confirm paternity and maternity.

### Evaluation of splicing abnormalities associated with intronic mutations

RNA was extracted from EBV-transformed lymphoblasts or lymphocytes with TriPure (Roche) (patients II-10 and V-12 and 2 controls) and retro-transcribed using RevertAid H Minus First Strand cDNA Synthesis Kit (Fermentas) with random hexamers. For patient II-10 and a control, PCR-amplification was performed using primers 5’-GAGGATTGGCTGACAAGAGC and 5’-CGTGGAATTATACCAGCCAAG. After agarose gel electrophoresis, bands were extracted using QIAquick® Gel Extraction Kit (QIAGEN) and purified amplicons were sequenced and compared to the reference sequence NM_004523.3. For patient V-12 and controls, amplicons were generated with 5’-GGTTTAGAAGAAATTACAGTACAC and 5’-TGACCCTTCCCAAAGTCAAC. They were cloned into the pCR-II TOPO vector (Invitrogen) according to the manufacturer’s protocol and the plasmids were transfected into JM109 competent bacteria (Promega, Madison, WI, USA) by heat shock. For each cDNA (patient and control), 16 clones were picked and inserts were PCR-amplified and sequenced using the same primers (above).

## Results

**Figure 3:**
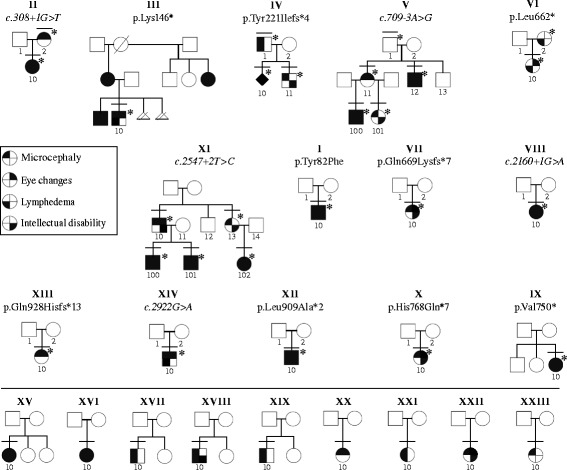
Pedigrees and phenotypes of screened patients. Upper panel, *KIF11* mutation-positive; lower panel, *KIF11* mutation-negative. Individuals with a bar, clinically examined; those with a number, sequenced. Patients with a star have a mutation.

Sequencing of the 22 coding exons of *KIF11* and their flanking splice-sites identified fourteen heterozygous mutations in the 23 index patients (Table 1 and Figure 2). All patients with family history (n = 6) had a mutation. These germline variants included two nucleotide substitutions and one single nucleotide deletion; all result in premature stop codons. Three splice-site alterations were also identified. The changes co-segregated with phenotype in the five families for which we had additional samples (n = 20) (Table 1 and Figure 3).

**Table 1:** **Clinical features of MCLMR patients presented in order of mutation position in** ***KIF11***

**Patient**	**Reference**	**Nucleotide variant NM_004523.3**	**Exon**	**Protein alteration**	**Inheritance**	**Microcephaly (SD)**	**Eye changes**	**Lymphedema**	**Intellectual disability**	**Additional clinical features**	**Phenotype or the same mutation described in**
**Patients with mutation**											
I-10	LE-401-10	*c.245A>T*	3	p.(Tyr82Phe)	*de novo*	−7.0	Chorioretinitis	Feet	Severe	Severe sensorineural hearing loss, proteinuria, upslanting palpebral fissures, broad nose with rounded tip, anteverted nares, long philtrum with thin upper lip and pointed chin	-
II-2	LE-09-2	*c.308+1G>T*	3	p.(Thr71Argfs*8)	AD	−4.0	Normal electroretinography but discrete narrowing of blood vessels	-	-	-	-
II-10	LE-09-10	*c.308+1G>T*	3	p.(Thr71Argfs*8)^*¶*^	AD	−3.0	Retinal dystrophy	Mild	Moderate	Scoliosis	-
III-10	LE-448-10	*c.436A>T*	5	p.(Lys146*)	AD	−4.4	Bilateral persistent hyperplastic primary vitreous, bilateral retinal detachment, bilateral glaucoma and inoperable retinal detachment right eye	Feet	-	Lensectomy, vitrectomy right eye and long philtrum	-
IV-1	LE-152-1	*c.630del*	6	p.(Tyr211Ilefs*4)	AD	−2.5	-	+	-	-	-
IV-10	LE-152-10	*c.630del*	6	p.(Tyr211Ilefs*4)	AD	−5.9	Retinal dystrophy	Mild	Moderate	Facial dysmorphism	-
IV-11	LE-152-11	*c.630del*	6	p.(Tyr211Ilefs*4)	AD	−5.9	Unknown, wears glasses	-	Moderate	Facial dysmorphism	-
V-1	LE-114-1	*c.790-3A>G*	8	p.(Val264Argfs*26)	AD	Within normal limits	-	-	-	-	-
V-11	LE-114-11	*c.790-3A>G*	8	p.(Val264Argfs*26)	AD	−2.2	Microphthalmos	-	-	-	-
V-12	LE-114-12	*c.790-3A>G*	8	p.(Val264Argfs*26)^*¶*^	AD	−2.9	Bilateral falciform retinal folds, microphthalmos, visual deficit and glaucoma	Feet as a child	Moderate	Sloping forehead	Young ID., *et al.*, 1987
V-100	LE-114-100	*c.790-3A>G*	8	p.(Val264Argfs*26)	AD	−6.6	Falciform retinal folds	Hands and feet	Moderate	Clinodactyly and facial dysmorphism	-
V-101	LE-114-101	*c.790-3A>G*	8	p.(Val264Argfs*26)	AD	−2.3	-	-	Mild	Left Club foot and dyslexia	-
VI-10	LE-103-10	*c.1985T>A*	15	p.(Leu662*)	AD	−5.0	Bilateral retinal degeneration	Feet	Mild	-	-
VI-2	LE-103-2	*c.1985T>A*	15	p.(Leu662*)	AD	-	-	Mild	Mild	-	-
VII-10	LE-411-10	*c.2005del*	16	p.(Glu669Lysfs*7)	*de novo*	−3v8	Retinal dystrophy	-	Moderate	Pachygyria	-
VIII-10	LE-125-10	*c.2160+1G>A*	16	Altered Splicing	*de novo*	−9.2	+	+	Moderate	Constipation and facial dysmorphism	-
IX-10	LE-406-10	*c.2244_2247dup*	17	p.(Val750*)	Sporadic	−3.0	Persistent hyperplastic primary vitreous posterior and retinal atrophy	Feet	Moderate	Facial dysmorphism	-
X-10	LE-413-10	*c.2304_2305del*	18	p.(His768Glnfs*7)	*de novo*	−5.4	Retinal dystrophy	-	Severe	-	Ostergaard P., *et a*l., 2012
XI-10	LE-08-10	*c.2547+2T>C*	18	Altered Splicing	AD	−7.3	Subtle atrophic pigment epithelial changes temporally from the optic disc on the right eye and atrophic changes prominent on the left eye	-	Moderate	-	Ostergaard P., *et a*l., 2012
XI-13	LE-08-13	*c.2547+2T>C*	18	Altered Splicing	AD	−4.4	Unknown	-	Moderate	Nasal speech and micrognathia	Ostergaard P., *et a*l., 2012
XI-100	LE-08-100	*c.2547+2T>C*	18	Altered Splicing	AD	−8.3	Microphthalmia, chorioretinopathy and retinal dystrophy	Feet	Moderate	Syndactyly, cardiopathy, pachygyria and facial dysmorphism	Casteels I., *et al*., 2001, Ostergaard P., *et al*., 2012
XI-101	LE-08-101	*c.2547+2T>C*	18	Altered Splicing	AD	−4.5	Bilateral corioretinal dystrophy	+	Moderate	-	Ostergaard P., *et a*l., 2012
XI-102	LE-08-102	*c.2547+2T>C*	18	Altered Splicing	AD	−3.5	-	Peri-oral	Moderate	Micrognathia, full nose tip, diathema of teeth and dysplastic ears	Ostergaard P., *et a*l., 2012
XII-10	LE-414-10	*c.2723dup*	19	p.(Leu909Alafs*2)	*de novo*	−5.5	Retinal dystrophy	+	Severe	-	Fryns JP., *et al*., 1995
XIII-10	LE-415-10	*c.2782_2783dup*	20	p.(Gln928Hisfs*13)	*de novo*	−3.8	Chorioretinal atrophy	-	Moderate	-	-
XIV-10	LE-104-10	*c.2922G>A*	20	Altered Splicing	*de novo*	−5.3	Myopic chorioretinal dysplasia	Hands and feet	-	Fairly broad nasal root, prominent ears and a narrow palate	-
**Patients without a mutation**											
XV-10	LE-87-10	No mutation	-	-	Sporadic	−6.9	Bulls eye maculopathy	Acute oedema at birth, resolved; bilateral lymphedema persisted	Severe	Hearing loss-bilateral sensorineural and facial dysmorphism	-
XVI-10	LE-88-10	No mutation	-	-	Sporadic	−8.1	Bilateral Falciform retinal folds	Mild, feet as a child	Moderate	Epilepsy and facial dysmorphism	-
XVII-10	LE-90-10	No mutation	-	-	Sporadic	−4.6	-	+	-	Lissencephaly, coronal craniosynostosis and ADHD	-
XVIII-10	LE-91-10	No mutation	-	-	Sporadic	−3.9	-	Feet and legs	Mild	Mild hypoplastic aortic arch, secundum atrial septal defects, mild motor delay, mild speech delay, ADHD and hyperlaxity	-
XIX-10	LE-121-10	No mutation	-	-	Sporadic	−3.0	-	Legs and face	Moderate	ADHD on methylphenidate treatment, hyperlaxity, bilateral Madelung deformity and egg allergy	-
XX-10	LE-393-10	No mutation	-	-	Sporadic	−5.0	Blind	Lower limbs	Severe	Cerebral atrophy, encephalopathy, cervicofacial lymphatic malformations, chylothorax, pleural effusion, hydrops fetalis and intestinal lymphangiectasia	-
XXI-10	LE-412-10	No mutation	-	-	Sporadic	−2.1	-	Feet	Moderate	Pectus excavatum, epicanthic folds and small ears	-
XXII-10	LE-417-10	No mutation	-	-	Sporadic	−4.4	Retinal dystrophy	-	Servere	Epilepsy	-
XXIII-10	LE-428-10	No mutation	-	-	Sporadic	−10.0	Chorioretinal dysplasia, myopia, nystagmus horizontale ODG	Mild	Severe	Arterial pulmonary hypertension, atrio-ventricular complete canal, motor delay, simplified gyral pattern, autistic behavior, ADHD, small for height (−4DS) and weight (−4DS), and facial dysmorphism.	-

+ = present, − = absent, AD = autosomal dominant, ¶ = mRNA tested, ADHD = Attention deficit hyperactivity disorder.

Among the 17 sporadic patients, we found eight with a mutation in *KIF11*: one nonsense, four frameshifts and two canonical consensus splice-site changes (Table 1) that we consider pathogenic; and one missense alteration that we regarded as probably pathogenic, but requiring further functional validation (Table 1). DNA from parents was available for 7 of the 8 sporadic patients with a *KIF11* mutation. All appeared to be *de novo* changes (100%) (Figure 3) absent in both parents, representing 41% (n = 7/17) of all our sporadic cases. In total, 30% of all the index cases studied (7/23) had *de novo KIF11* mutations.

**Figure 4:**
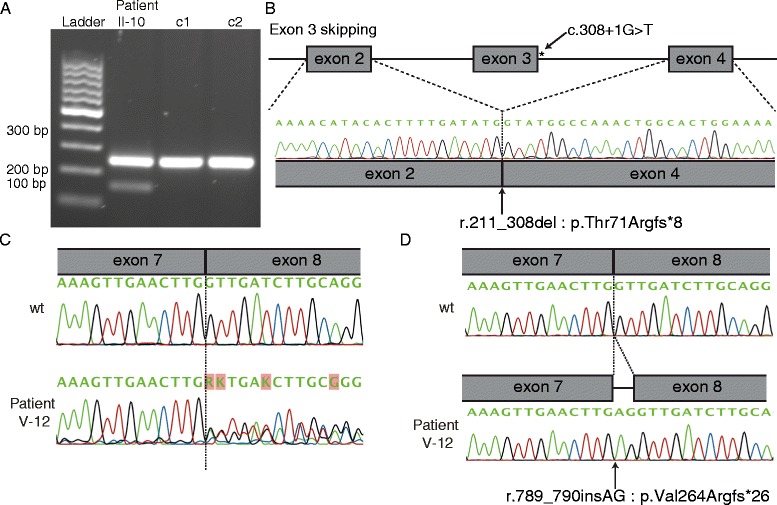
Effect of splice site mutations. **(A)** RT-PCR using primers in exon 2 and 5 on cDNA from patient II-10 (*c.308+1G>T*) and two controls (c1 and c2). Wild-type amplicon, 250 bp; mutant, 152 bp. **(B)** (Sequencing of lower band unraveled skipping of exon 3 resulting in a premature stop-codon (r.211_308del: p.Thr71Argfs*8). **(C)** Sequencing of cDNA of patient V-12 (*c.790-3A>G*) for an amplicon covering exon 7/8 splice site revealed double-sequence. **(D)** Sequencing of cloned amplicon fragments revealed insertion of 2 nucleotides (AG) from the intronic acceptor site (r.790_791insAG) in mutant clones. This leads to a premature stop-codon (p.Val264Argfs*26).

The mutations detected most likely result in loss of function of *KIF11* due to nonsense-mediated mRNA decay or premature truncation of the protein (n = 13). Lymphoblast RNA from a patient with a splice-site alteration (Patient II-10: *c.308+1G>T*) showed skipping of exon 3 (r.211_308del) (Figure 4A), leading to a change in reading frame (p.Thr71Argfs*8) (Figure 4B). The mutant transcript was expressed at a lower level than the wild-type allele, suggesting mRNA loss by nonsense-mediated mRNA decay. In patient V-12, the splice-variant (*c.790-3A>G*) generates a new AG-acceptor site-like sequence. In lymphocytes, this led to the insertion of 2 nucleotides from the intronic splice site (r.789_790insAG) into the mRNA (Figure 4C and D), causing a reading frame shift and appearance of a premature stop codon 26 amino acids after (p.Val264Argfs*26). We identified only 1 mutation leading to a heterozygous amino acid substitution: Tyr82Phe (Patient I-10). The substitution occurs at a highly conserved amino acid position in the kinesin motor domain from fish to human being. It is predicted to alter protein function by PolyPhen-2, SIFT and Mutation Taster 2.0. The change is absent in the 1000Genomes, ESP6500, and GoNL databases.

Taking into account all the patients found to carry a mutation in this study (n = 26), as well as the 61 already reported [6-10], microcephaly was present in 79/87 patients (91%), eye anomalies in 63/87 patients (72%), intellectual disability in 58/87 (67%) and lymphedema in 41/87 patients (47%). Average SD for microcephaly was −5,1 (ranging from −1,1 to −9,5), on the basis of 64 patients with published data. None of the clinical signs was fully penetrant. Atypical features including cerebral and cardiac anomalies were present in 48 patients (55%). Taking all index patients with mutations into account (n = 52), sporadic cases represent 42% (22/52). It has been proven in 73% (16/22) of them that the mutation appeared *de novo*.

The nine *KIF11* mutation-negative sporadic patients were included in *KIF11* mutation screening due to the presence of signs and symptoms corresponding to MCLMR. However, certain patients showed additional features, not described for MCLMR: hydrops fetalis and lymphangiectasia (1 patient: XX-10), motor delay (2 patients: XVIII-10, XXIII-10), and Madelung deformity (1 patient: XIX-10). These patients probably reflect the clinical and genetic variability of microcephaly syndromes.

## Discussion

We discovered 14 *KIF11* mutations, 12 of which are novel, in 26 MCLMR patients (Figure 2). A mutation was detected in all patients with a family history of MCLMR, similar to previously reported series [6-10]. We also discovered a heterozygous germline *de novo* mutation in 7 sporadic patients and in 1 patient without family history of MCLMR, but for whom no parental DNA was available for testing. Thus, 87,5% of our sporadic patients with a *KIF11* mutation had a proven *de novo* mutation. This represents 41% (7/17) of sporadic index patients in this study. Taking into account all index patients with a *KIF11* mutation, sixteen sporadic patients have been reported to have a *de novo* mutation (7 in the current study, and 9 in previously published works), indicating that *KIF11* has a high *de novo* mutation rate: 16 *de novo*/52 mutation-positive index patients; i.e. 31% across studies. Several *de novo* mutations have also been identified in other genes mutated in microcephaly (i.e. CTNNB1 [20] and TUBA1A [21]) and in primary lymphedema, such as *SOX18*, *FOXC2* and *VEGFR3* [22-24].

No *KIF11* mutation-negative familial cases were identified; however, about half of the sporadic cases were *KIF11* mutation-negative. These may be explained by several hypotheses: (1) Deletions or deep-intronic mutations in *KIF11*, which were not assessed in the study; (2) Somatic/mosaic *KIF11* mutations that are not detectable in the blood by Sanger sequencing: mosaicism as a cause of MCLMR can be investigated by sequencing tissue and blood-DNA using targeted deep sequencing; (3) Existence of a mimicking disorder: many different genes can cause microcephaly, including those implicated in autosomal recessive primary microcephaly and syndromic microcephaly [25], or primary lymphedema [26]. The presence of motor delay in patients XVIII-10 and XIII-10 may suggest Cohen syndrome (OMIM 216550) [27]. The Madelung deformity and hydrops fetalis observed in two other patients may also be indicative of a distinct syndrome, as they have never been described in MCLMR. Eight out of nine of our negative cases had microcephaly and lymphedema; (4) Novel genetic cause for MCLMR in mutation-negative patients. Overall, our results suggest that *KIF11* is the major causative gene for MCLMR, with no evidence of locus heterogeneity in familial cases.

The most frequent clinical signs and symptoms in patients with a *KIF11* mutation are microcephaly (91%), eye anomalies (72%) and intellectual disability (67%) (Figure 3 and Table 1). MCLMR patients may have a structurally abnormal brain *i.e*. pachygyria (Table 1: Patients VII-10 & XI-100) [9]. Lymphedema is present in 47% of mutation-positive patients (Table 1).

Although none of the individual symptoms is fully penetrant, the overall penetrance of MCLMR is high (83/87; 95%). Only 4 asymptomatic mutation carriers have been identified amongst 87 individuals tested. Patients in the same family (Family II, IV, V, VI, and XI), with the same mutation may however have different phenotypes, demonstrating variable expressivity.

The 44 mutations identified so far in *KIF11* (nine missense, twenty-five premature stop codons and ten splice-site changes) are scattered across the length of gene (Figure 2). There are no clear phenotypic differences between missense mutation carriers and those with premature stop codon mutations. As the most 5’ premature truncation codon occurs at the very 5’ end (amino acid 47), and lymphoblasts had a significantly lower expression of the mutant allele of patient II-10 (*c.308+1G>T*), all mutations most likely cause loss of *EG5* function.

*KIF11* mutations have pleiotropic effects with variable penetrance. One possible explanation could be the occurrence of somatic second-hit mutation(s), as we have proven for glomuvenous malformation [28,29] and mucocutaneous venous malformation [30]. Random somatic changes inevitably occur in the cells of all individuals, and result in a double-hit situation in some cells in patients with a germline mutation. The resulting phenotypes would depend on the types and developmental stages of cells affected.

There are nine amino acid substitutions identified in MCLMR patients. One of them affects the initiating methionine at position 1 (p.Met1Thr) most likely causing non-translation of *KIF11*. Seven of the remaining eight amino acid substitutions occur in the kinesin motor domain (p.Tyr82Phe, p.Phe144Leu, p.Arg221Gly, p.Arg234Cys, p.Ser235Cys, p.His244Tyr, and p.Glu344Lys) and the last one in the BimC Box (p.Arg944Cys), implicated in multimerization (Figure 2). Both domains have been shown to be important for *KIF11* function during mitosis [11]. The kinesin motor domain contains amino acids important for tertiary structure. Arg234 makes a salt bridge with Glu270 to close the active site essential for ATP hydrolysis [31]. Substitution of Arg234 for a cysteine likely perturbs closure of the pocket and ATP hydrolysis. The impact of Ser235Cys is likely similar due to its vicinity to Arg234. In addition, the serine-to-cysteine change would affect polarity and thus modify the stabilization of a molecule of water in the nucleotide-binding pocket. Moreover, in the 3D structure (PDB ID: 3hqd), Arg221 is located in front of Ser235. Substitution for glycine could influence the structure of the nucleotide-binding pocket. Tyr82, Phe144 and Glu344 are located in an alpha-helix and His244 in a central beta-sheet in the kinesin domain. It is unclear whether these mutations, and the BimC Box mutation, affect stability, structure and/or function.

*In vitro* mutagenesis (Pro131Ala, Thr926Ala and Ser1033Ala) showed that the N-tail and the BimC domain are important for intra-cellular localization of the protein [32]. Thr926 of the BimC domain is phosphorylated by CDK1 (Cyclin-dependent kinase 1), whereas Ser1033 is partially phosphorylated by NEK6 (Never in mitosis gene a -related kinase 6). Phosphorylation of these sites is important for the mitotic function of EG5 [33,34]. Depletion of *KIF11* causes mitotic arrest. This can be rescued by re-expression of the protein, but not by a Thr926Ala mutant, which lacks the ability to interact with microtubules [33]. The Arg944Cys mutation likely causes dysfunction of this same domain.

EG5 is a plus end-directed microtubule motor. It contributes to the establishment and maintenance of bipolar spindles during mitosis and meiosis that push the poles apart [35]. This is required for the separation of duplicated centrosomes. EG5 is considered as a slow kinesin, which slows down the spindle separation during mitosis. It is dispersed in the cytoplasm during the interphase, regroups at the spindle poles during prophase and stays alongside the spindle in metaphase. In mice, Eg5 is expressed in proliferative tissues. Disruption by homozygous deletion of *Kif11* in mice resulted in embryonic lethality due to defective implantation in the uterine wall [36]. Microcephaly, ocular anomalies and œdema were not reported in heterozygous embryos. Morpholino-based knock-down of *Kif11* also led to embryonic lethality, or severe œdema and circulation defects [37]. Inhibition of EG5 by dimethylenastron (DMN) or ispinesib diminished endothelial cell proliferation (HUVEC, hCMEC/D3 and LEC) and embryonic angiogenesis [37].

EG5 disruption is implicated in neuronal development and the phenotype observed in MCLMR adds *KIF11* to the long list of genes implicated in intellectual disability. Inhibition of EG5 in cultures of synaptic neurons led to faster growth of axons, with an abnormal maturation [38]. Intellectual disability due to EG5 dysfunction could thus be explained by neuronal inability to grow and/or to connect correctly. The etiopathogenesis of the eye anomalies could be due to defective neurogenesis or angiogenesis.

EG5 could cause lymphedema by a microtubule-dependent function in trafficking the major lymphangiogenic tyrosine kinase receptor, VEGFR3, in endocytotic vesicles [39]. On the other hand, it could be that the endothelial cell function of EG5 is independent of the kinesin-motor domain, as is the case for KIF26A (kinesin family member 26A), KIF4 (kinesin family member 4A/B) and MAP2 (microtubule-associated protein 2) [40-42]. In fact, EG5 plays also a role in protein translation [43]. The expression of *KIF11* in LECs is regulated by FOXC2, a major lymphangiogenic transcription factor [44]. It is however not known if MCLMR patients have lymphatic hyperplasia as observed in FOXC2-mutated patients (lymph and venous reflux and failure or absence of lymphatic and venous valves) or hypoplasia as seen in VEGFR3-mutation caused Nonne-Milroy lymphedema [45,46]. Lymphoscintigraph of one MCLMR patient showed no significant main-tract filling suggesting a peripheral lymphatic-vessel dysfunction [6].

## Conclusions

*KIF11* mutations are identified in all familial cases of MCLMR. *De novo* mutation rate is high for *KIF11*: 16 out of 22 (73%) sporadic cases. Mutations most likely cause loss of EG5 function. There is no genotype-phenotype correlation. The phenotype of the *KIF11* mutation-negative sporadic patients may be due to non-detected germline or mosaic *KIF11* mutations and/or defects in (a) gene(s) causing (a) mimicking disorder(s), as microcephaly and primary lymphedema are genetically highly heterogeneous. A better elucidation of the pathophysiological mechanisms is needed to enable development of targeted therapies for MCLMR.

### Availability of supporting data

The mutations were submitted to the Vascular Anomaly and Lymphedema Mutation Database (www.icp.ucl.ac.be/vikkula/VAdb).

## Abbreviations

MCLMR: Microcephaly with or without chorioretinopathy, lymphedema, or mental retardation
KIF11: Kinesin family member 11
CDMMR: Chorioretinal dysplasia, microcephaly and mental retardation syndrome
MLCRD: Microcephaly, lymphedema and chorioretinal dysplasia syndrome
FEVR: Familial exudative vitreoretinopathy syndrome
EBV: Epstein-barr virus
SOX18: SRY (sex determining region Y)-box 18
FOXC2: Forkhead box C2 (MFH-1, mesenchyme forkhead 1)
VEGFR3: Fms-related tyrosine kinase 4
CTNNB1: Catenin beta 1
TUBA1A: Tubulin alpha 1a
